# The Putative Link Between Omodysplasia and Treatment-Resistant Schizophrenia: A Complex Clinical Presentation of a Rare Genetic Disorder

**DOI:** 10.7759/cureus.66699

**Published:** 2024-08-12

**Authors:** Soumitra Das, Sangam Giri, Darshini B Shah, Palak A Fichadia, Mukund Rao, Shyam Ravilla

**Affiliations:** 1 Psychiatry and Behavioral Sciences, Western Health, Victoria, AUS; 2 Medicine, Kathmandu Medical College, Kathmandu, NPL; 3 Medicine, Gujarat Cancer Society (GCS) Medical College, Hospital and Research Center, Ahmedabad, IND; 4 Psychiatry, Icahn School of Medicine at Mount Sinai, New York City, USA; 5 Psychiatry and Behavioral Sciences, Northern Health, Victoria, AUS

**Keywords:** schizo-phrenia, anti-psychotics, psychosis, schizophreniform disorder, omodysplasia

## Abstract

Genetic and metabolic disorders present unique challenges in understanding the pathophysiology and outcomes of specific symptoms and presentations due to their broad spectrum of manifestations and etiologies.

In this case report, we have studied a 26-year-old who was diagnosed with omodysplasia, a rare form of skeletal dysplasia. She exhibits atypical symptoms of psychosis and was diagnosed with schizophreniform disorder at an early age. Various antipsychotic medications were administered; however, minimal to no improvement was noted in the symptoms. On the contrary, she reported adverse effects to some antipsychotics. She continued to exhibit delusions and hallucinations and showed clinical improvement after treatment with olanzapine. Her clinical course was further complicated by the presence of borderline personality traits, which went unnoticed earlier.

Here, we would like to highlight the course of her symptoms, the different treatments administered, and the possible link between omodysplasia and treatment-resistant schizophrenia.

## Introduction

The biological pathway linking an individual’s genes to a behavioral abnormality is complex and poorly understood. Genes can affect physical, physiological, or neuronal development. Rare genetic diseases can present with complex neurobehavioral abnormalities. It is important to diagnose these patients, discuss prognosis, and identify potential gene therapies if any [1].

An uncommon short-limb skeletal dysplasia known as autosomal recessive omodysplasia (GPC6-related) is brought on by biallelic mutations in the GPC6 gene. Rhizomelic stunted height, limited joint mobility in the elbow and knee, and craniofacial deformities are all symptoms of the condition. The lower and upper limbs are also significantly impacted [2]. 

Other organ systems can also be involved in omodysplasia and are linked to congenital heart illness, like coarctation of the aorta, atrial septal defect, mitral valve prolapse, patent ductus arteriosus, forehead prominence with central hemangioma and large low-set ears. Mental retardation has also occasionally been reported with this disease, which may make history-taking more challenging [3].

In addition, according to the neurodevelopmental model of schizophrenia, structural and functional changes in the brain occur during the perinatal and intrauterine periods, as well as in childhood and the early adolescent years. As a result of both hereditary and environmental variables, the possible neuropsychological link between schizophrenia spectrum disorders and patients with skeletal dysplasias or congenital anomalies has also been postulated [4,5].

Here, we present a case of omodysplasia who presented with long-standing atypical symptoms of psychosis, which were difficult to treat secondary to side effects and doubtful compliance. To the best of our knowledge, there is no published scientific literature regarding the neurobehavioral manifestations of omodysplasia. 

## Case presentation

A 26-year-old unmarried female had a long-standing diagnosis of omodysplasia, which was identified during her early childhood. At the age of 13, she experienced the onset of psychosis and was diagnosed with schizophreniform disorder. Over time, she underwent trials of various antipsychotic medications but achieved limited response. Initial treatment attempts using quetiapine and olanzapine were ineffective. Furthermore, she developed adverse effects when administered low doses of lurasidone, risperidone, and aripiprazole. 

She occasionally believed she was pregnant, although this claim was contradicted by medical examinations and testing, affirming it to be a product of her own imagination. The patient exhibited difficulties in articulating her paranoid thoughts, yet her distress regarding personal safety was objectively evident. Gradually, she began reporting experiences of hypnagogic hallucinations and a narrower range of emotions. She frequently expressed confusion and, on several occasions, claimed to detect the scent of human flesh, as though implying involvement in cannibalistic activities by those around her. She misinterpreted physical symptoms as indicative of multiple sclerosis and attributed these sensations to malevolent spiritual forces or the "evil eye."

Furthermore, she believed that her thoughts and feelings were accessible to others and that supernatural entities communicated with her through the medium of traffic lights. During her most recent interaction with our public mental health team, the patient was initiated on chlorpromazine, and discussions regarding a potential trial of clozapine were initiated. Subsequently, she reported some improvement in her psychotic symptoms upon the administration of a low-dose olanzapine depot, resulting in a reduction in her paranoia.

Additionally, an inclination towards splitting behavior manifested in the patient’s interactions with the treatment team. She encountered significant difficulties in her relationship with her mother, and she displayed an eagerness to impress and minimize conflicts, which did not appear to be driven by psychosis. She also exhibited a pattern of frequently missing scheduled appointments, demonstrating enhanced engagement with the registrar while displaying less involvement with the consultant or her primary clinician.

Based on her clinical presentation and history, the patient received a diagnosis of schizophrenia alongside borderline personality traits.

## Discussion

Omodysplasia-1 (OMOD1) is a rare autosomal recessive skeletal dysplasia characterized by distinct physical features, such as shortening and distal tapering of the humeri and femora, leading to a club-like appearance. Additional facial characteristics include a prominent forehead, frontal bossing, short nose with a depressed broad bridge, short columella, anteverted nostrils, long philtrum, and small chin. Chromosome analysis typically reveals a paternally inherited paracentric inversion, inv(15)(q13q21.3) [6].

One specific genetic mutation found in omodysplasia involves Glypican, a family of Heparan sulfate proteoglycans (HSPGs). HSPGs are known to play a role in regulating essential developmental events, such as morphogen gradient formation, nervous system development, and the stem cell niche [7]. 

In the case presented, the patient exhibited long-standing psychotic symptoms that have been difficult to treat. Sensitivity to different medications may be attributed to impaired brain development [8]. The patient has shown a positive response to olanzapine depot, which raises the possibility of non-compliance with oral medications. Recent interactions with the treating team have suggested a borderline personality structure.

While there is no reported neurobehavioral variant specific to omodysplasia, it is important to note that rare genetic disorders can present complex behavioral issues. Some reports have indicated developmental delay, lack of speech, hypersomnolence, poor feeding, and epilepsy as associated features [1,9]. Involvement of the central nervous system in omodysplasia can manifest as impaired cognition, behavioral disorders, epileptic seizures, difficulties in language and speech, sleep problems, and hyperactive and/or aggressive behavior [9].

This case underscores the challenges faced in managing rare genetic diseases. These challenges can arise due to side effects of medications, complicated family dynamics, and potential neuro-cellular changes resulting from unknown mechanisms. Proper and timely genetic analysis, along with the involvement of a clinical geneticist, can aid in prognosis and facilitate the development of practical and appropriate treatment plans.

It is crucial to recognize the unique complexities presented by rare genetic disorders and address them through interdisciplinary collaboration, comprehensive genetic analysis, and tailored treatment approaches for improved outcomes.

## Conclusions

Hence, it is crucial to identify co-morbidities associated with psychiatric disorders as this can complicate the treatment modality and even change it. We as physicians need to be more vigilant in identifying genetic components of psychiatric disorders, especially ones with treatment resistance, and include these rare modalities in our list of differentials. 
